# Midtrimester Evaluation of Fetal Heart with Fetal Heart Quantification (FetalHQ) Related to Maternal Pathology

**DOI:** 10.3390/jcm15041352

**Published:** 2026-02-09

**Authors:** Stefano Raffaele Giannubilo, Camilla Grelloni, Alessandro Cecchi, Elisa Carboni, Giuseppe Maria Maruotti, Sara Mannolini, Alessia Maria Merone, Maria Terrone, Andrea Ciavattini

**Affiliations:** 1 Department of Clinical Sciences, Obstetrics and Gynecology Section, Università Politecnica Delle Marche, 60123 Ancona, Italy; c.grelloni@pm.univpm.it (C.G.); a.ciavattini@univpm.it (A.C.); 2 Centro Unico Regionale SODS, Diagnosi Prenatale di II Livello, 60025 Loreto, Italy; alessandro.cecchi@sanita.marche.it (A.C.); elisa.carboni@sanita.marche.it (E.C.); 3 Gynecology and Obstetrics Unit, Department of Public Health, University of Naples Federico II, 80138 Naples, Italy; giuseppemaria.maruotti@unina.it (G.M.M.); sara.mannolini@gmail.com (S.M.); alessia.merone@gmail.com (A.M.M.); maria_terrone@live.it (M.T.)

**Keywords:** echocardiography, fetalHQ, obesity, diabetes, FGR

## Abstract

**Background**: Echocardiography currently represents the gold standard for the anatomical and functional assessment of the fetal heart by experienced operators. FetalHQ (VolusonTM) provides a semi-automated speckle-tracking analysis of the fetal heart with promising results for the reliable assessment of cardiac remodeling, specifically of size, shape, and contractility. **Methods**: We conducted a retrospective study comparing 108 controls, 119 obesity (BMI ≥ 30), 69 pre-pregnancy diabetes mellitus (DM), 41 gestational diabetes mellitus (GDM), and 37 early fetal growth restriction (FGR) cases (19+0–22+6 weeks). FetalHQ was utilized during midtrimester echocardiographic exams to evaluate fetal myocardial thickness and left ventricular mass. **Results**: Myocardial thickness was increased in DM (median 0.21 [IQR 0.17–0.23] cm) vs. controls (0.17 [0.15–0.21] cm; *p* < 0.01) and reduced in FGR (0.13 [0.11–0.18] cm; *p* < 0.01). Left ventricular mass increased in obesity (0.98 [0.62–1.19] g) and DM (1.12 [0.94–1.17] g) vs. controls (0.73 [0.56–0.97] g; both *p* < 0.01). **Conclusions**: Fetal cardiac remodeling, especially myocardial thickness and left ventricular mass adaptations, is detectable in fetuses from high-risk pregnancies starting from the second trimester. Advanced speckle-tracking techniques, such as fetalHQ, provide valuable early risk stratification and may support the need for systematic fetal cardiac screening and monitoring during gestation in selected groups of patients.

## 1. Introduction

The human heart is one of the first organs to form and function during embryogenesis, and its development during intrauterine life is a complex process driven by multiple genetic, epigenetic, and morphodynamic factors [1]. Cardiomyocytes are subjected to proliferation, differentiation and hypertrophy during intrauterine life [2].

Fetal echocardiography is an advanced ultrasound examination performed by experienced operators in referral centers in pregnancies at high risk of fetal congenital heart disease. In high-risk referral practice, fetal echocardiography is frequently driven by screening-based indications and soft markers, with the majority of examinations (88.5%) remaining normal [3]. The clinical purpose is to provide a global assessment of the fetal heart, and, when an abnormal finding is detected, to counsel parents about diagnosis, long-term implications, and neonatal outcome, as well as to discuss treatment options [4].

Modern functional sonographic techniques—including Tissue Doppler Imaging (TDI), Myocardial Performance Index (MPI), Speckle-Tracking Echocardiography (STE), and four-dimensional Spatio-Temporal Image Correlation (STIC)—have been progressively introduced into echocardiography over the last two decades. TDI and MPI were increasingly applied in the early 2000s as instruments for the detailed evaluation of fetal cardiac function, while, by the last decade, two-dimensional STE was standardized to assess ventricular deformation throughout the cardiac cycle in fetuses. Three- and four-dimensional STIC technology performs a volumetric acquisition of the fetal heart and its modifications during the cardiac cycle, enhancing both anatomical and functional analysis [5,6,7,8,9,10,11].

With the continuous advancement of sonographer expertise and ultrasound technology, the role of fetal echocardiography has evolved: once primarily a tool for diagnosing congenital heart defects (CHDs), it is now progressively used to evaluate fetal cardiac remodeling, including anatomical structural changes and myocardial functional alterations [12].

Functional changes in the heart may represent early manifestations in the fetal adaptive response to various stressors, potentially leading to circulatory system dysfunction [13,14]. Therefore, the evaluation of fetal cardiac structure and function can provide valuable information for the diagnosis and monitoring of pregnancy complications [8].

Cardiac remodeling is defined by international guidelines as an alteration in the size, shape, or function of the heart in response to an insult [12]. In the fetal period, such remodeling has been associated not only with CHDs but also with other conditions, including conception via assisted reproductive technologies, fetal growth restriction (FGR), maternal pre-pregnancy diabetes (DM), and exposure to certain medications [12].

These findings have stimulated growing interest concerning maternal factors and obstetric conditions that may affect myocardial tissue, leading to an increase in echocardiographic studies of fetal cardiac remodeling and function during the late stages of pregnancy.

Most data on this topic come from studies with small populations designed to compare fetuses exposed to common maternal conditions (e.g., pre-gestational or gestational diabetes, obesity, autoimmune diseases) with low-risk controls, adjusting for gestational age, fetal size, and maternal comorbidities. This is mostly because advanced fetal cardiac evaluation techniques (TDI, STE, and STIC) are usually performed in single-centers due to expertise availability which limits patient recruitment [15,16,17,18,19,20,21]. Most of these works consistently highlighted significant fetal cardiac remodeling in pregnancies complicated by diabetes, obesity, or autoimmune diseases. These structural and functional alterations observed in utero often persist after birth, which could impact on neonatal cardiac performance in the future [15,16,17,18,19,20,21].

Fetal heart quantification (fetalHQ) is a novel instrument for the semi-automated quantitative assessment of fetal heart size, morphology, and functionality using STE of the endocardium [22], with moderate inter-observer and intra-observer reproducibility [14].

Several studies recently introduced fetalHQ during the late second and third trimester echocardiography scan, showing an optimal detection rate performance for cardiac remodeling in high-risk pregnancies [10,22,23,24,25].

The aim of this study was to compare fetal cardiac remodeling using the fetalHQ tool (General Electric (GE) Voluson™ E10 ultrasound device equipped with a trans-abdominal C2-9-D convex probe (2-9 MHz) as well as the software tool fetalHQ^®^, GE Healthcare, Zipf, Austria) during second-trimester echocardiography, across two pre-pregnancy maternal conditions (DM and obesity), gestational diabetes (GDM), and FGR. FGR arises from complex etiologies, with placental dysfunction predominant (e.g., impaired spiral artery remodeling), alongside maternal factors (hypertension, diabetes), fetal issues (aneuploidy, infections), and placental pathology (malperfusion). Unlike obesity/DM/GDM—which are maternal metabolic disorders—early FGR (<32 weeks) in this cohort aligns with placental-mediated mechanisms, justifying its inclusion as a comparator for fetal cardiac effects.

## 2. Materials and Methods

### 2.1. Study Population

We conducted a retrospective, single-center cohort study of pregnant patients at the Clinic of Obstetrics and Gynecology of the Polytechnic University of Marche in collaboration with the outpatient facility Regional Center for Prenatal Diagnosis (Loreto, Marche, Italy), between 1 January 2023, and 31 December 2024. All patients underwent fetal echocardiography during their midtrimester ultrasound as part of their prenatal care or because their gestations were considered at high risk for fetal congenital heart disease (CHD). Echocardiography scans were performed according to our center’s standard protocol, which aligns with guidelines of the International Society of Ultrasound in Obstetrics and Gynecology (ISUOG) [26,27,28]. We included all single pregnancies that ended in the birth of a live baby without malformations. Pregnancies with genetic alterations, severe fetal malformations, fetal demise, and those complicated by preeclampsia and preterm birth/premature rupture of membranes were excluded. Gestational age was determined by first-trimester ultrasound examination between 12+0 and 13+6 weeks of gestation, according to the measure of the fetal crown–rump length [29]. Groups were defined by primary obstetric complication: obesity (BMI) (pre-pregnancy), diabetes mellitus (DM) (pre-gestational), gestational diabetes mellitus (GDM), early fetal growth restriction (FGR); controls were randomly selected from low-risk exams (MedCalc random sampling). Comorbidities were recorded but assignment was mutually exclusive per clinical indication—no double-counting of patients across pathological groups. Patient overlap between high BMI and GDM groups was possible but controlled through group assignment based on primary diagnosis. Gestational diabetes mellitus (GDM) was diagnosed in women who underwent an oral glucose tolerance test based on clinical indication and met the diagnostic criteria of the American Diabetes Association [30,31]. Maternal obesity was established when patients had a pre-gestational body mass index (BMI) ≥ 30 kg/m^2^ [32]. Fetal growth restriction (FGR) was diagnosed according to international guidelines (Delphi Consensus) [33] with criteria for early FGR (below 32 weeks) when either one solitary criterion or two or more contributory criteria were present in the absence of congenital anomalies. Solitary criteria included an estimated fetal weight (EFW) or abdominal circumference below the 3rd percentile for gestational age, or absent end-diastolic flow in the umbilical artery (UA-AEDF). Contributory criteria comprised an EFW or abdominal circumference below the 10th percentile in combination with an umbilical artery pulsatility index (UA-PI) above the 95th percentile or a mean uterine artery pulsatility index above the 95th percentile. The control group (n = 108) was prospectively identified from a consecutive pool of midtrimester fetal echocardiograms (19+0 to 22+6 weeks) performed at the same center in the same period. Low-risk criteria included: singleton pregnancy, maternal BMI 18.5–29.9 kg/m^2^, no pre-gestational/gestational diabetes, no hypertension, no FGR, no smoking, and no assisted reproductive technology (ART). Selection used random consecutive sampling ** (MedCalc v.20 random number generator, seed = 12,345) among exams with optimal fetalHQ image quality (no rib shadows, frame rate ≥ 80 Hz, clear 4-chamber view). Group-level matching targeted median GA of 21–23 weeks (no individual 1:1 propensity matching, as per similar fetalHQ studies [14]).

### 2.2. Instruments and Fetal Echocardiography Protocol

All patients underwent fetal cardiac examination between 19 and 22+6 weeks using two-dimensional ultrasound on a GE VolusonTM E10 ultrasound system (General Electric Healthcare Ultrasound, Zipf, Pfaffing, Austria) equipped with a transabdominal 2–9 MHz broadband convex probe (C2-9-D). All fetal echocardiographic exams were conducted by two experienced operators (A.C. and E.C.). Patients were positioned supine, and a routine obstetrical scan was performed to assess overall fetal condition, during which biparietal diameter (BPD), head circumference (HC), abdominal circumference (AC), and femur length (FL) were measured to estimate fetal weight and confirm gestational age. Fetal heart quantitative analysis was performed through fetalHQ software for STE, integrated into GE VolusonTM machines. Image acquisition followed the protocol recommended by VolusonTM [22,34,35]:

- Step 1: Acquisition of a high-quality 4-chamber view cine-loop by setting the machine to fetal heart preset (medium/low harmonic, narrow angle, low depth, frame rate: min. 80 Hz). The image was optimized with the heart centered in the image, no visible rib shadows, and heart orientation with the left ventricle on the left if the apex was at the top or clockwise before the right ventricle.
- Step 2: Drawing an M-mode line from the apex to the lateral base of the right ventricle and selecting a representative cardiac cycle (end-systolic and end-diastolic phases).
- Step 3: Define left or right ventricular endocardial contours of each cardiac phase by placing tracking on the endocardial borders

**Figure 1:**
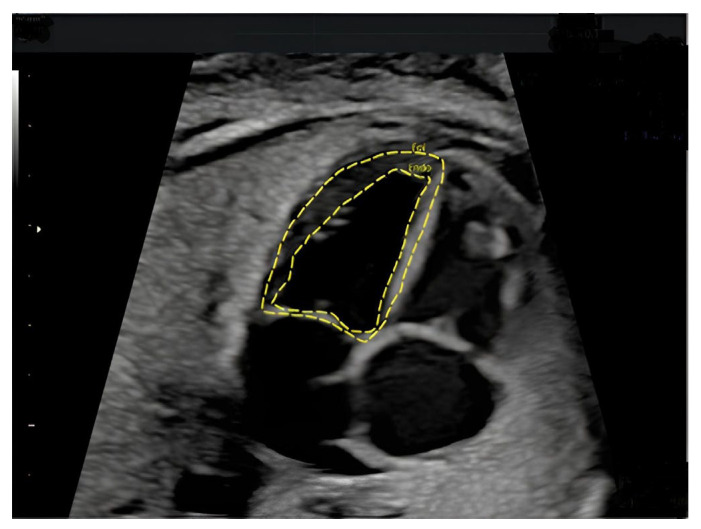
Four-chamber view with calipers positioned for left ventricular end-systolic endocardial and epicardial tracing.

At this point, the software automatically divides the left and right ventricles of the fetal heart into 24 segments and analyzes the quantitative indexes thickness, size, contractility and functionality of both ventricles throughout the cardiac cycle using speckle-tracking algorithms. Figure 1 shows an example of how calipers should be placed in four-chamber view to estimate fetal left ventricular mass and myocardial thickness at 25+0 weeks gestation. We recorded, for each patient, age, BMI, gestational weeks at the exam, DM, GDM, EFW, myocardial thickness and fetal left ventricular mass. Data on pregnancy outcomes were obtained from the electronic medical records, whether delivery occurred at our center or at other regional hospitals. All datasets were either analyzed or directly supervised by one of the authors (A.C.).

### 2.3. Statistical Analysis

Variables with a normal distribution are presented as the mean ± standard deviation (SD), while variables that did not follow a normal distribution are expressed as medians (interquartile range (IQR)).

Student’s *t*-test was used for comparing normally distributed continuous variables, while chi-square or Fisher’s exact test was applied for categorical variables as appropriate.

Kruskal–Wallis’ omnibus test (>2 groups) was followed by Dunn’s multiple comparison test with Bonferroni’s correction (α = 0.05/6 pairwise comparisons). A significant omnibus test was required for post hoc testing. Differences were considered statistically significant with *p* < 0.05. Statistical processing was performed with MedCalc^®^ v.20 (MedCalc Software Ltd., Gent, Belgium).

As a retrospective observational study using de-identified clinical data from electronic records, no prospective planning or informed consent was required per Italian/EU regulations (D.Lgs 196/2003, GDPR Art 89). Institutional Review Board approval was obtained; this is standard for single-center chart reviews in obstetric research.

## 3. Results

The final study population included 119 obese patients (BMI group), 69 with pre-gestational diabetes mellitus (DM group), 41 with gestational diabetes mellitus (GDM group), and 37 with early fetal growth restriction (FGR group). We also enrolled 108 patients with normal pregnancies, matched for gestational age, as a control group (Controls). Maternal and pregnancy characteristics are shown in Table 1. Patients’ age and weeks of gestation at echocardiographic assessment did not differ significantly between the Controls and case groups (BMI, DM, GDM and FGR). BMI was significantly higher in the obese (BMI) and GDM groups, whereas it was lower in the DM and FGR groups. Estimated fetal weight (EFW) was also comparable between controls and cases, with the FGR group showing a slightly lower mean weight that did not reach statistical significance.

Reported *p*-values (Student’s *t*-test: Controls 22.4 ± 3.78 vs. BMI 21.5 ± 2.3, *p* = 0.029; DM 20.4 ± 1.64, *p* = 0.013) reflect non-normal distributions (Kolmogorov–Smirnov *p* < 0.05 due to wide IQR in Controls). Non-parametric analysis shows overlapping medians: Controls 22.0 [20.5–24.0], BMI 21.5 [20.0–22.5], DM 20.5 [19.5–21.0] weeks (Kruskal–Wallis *p* = 0.12; Dunn post hoc all *p* > 0.05).

**Figure 2:**
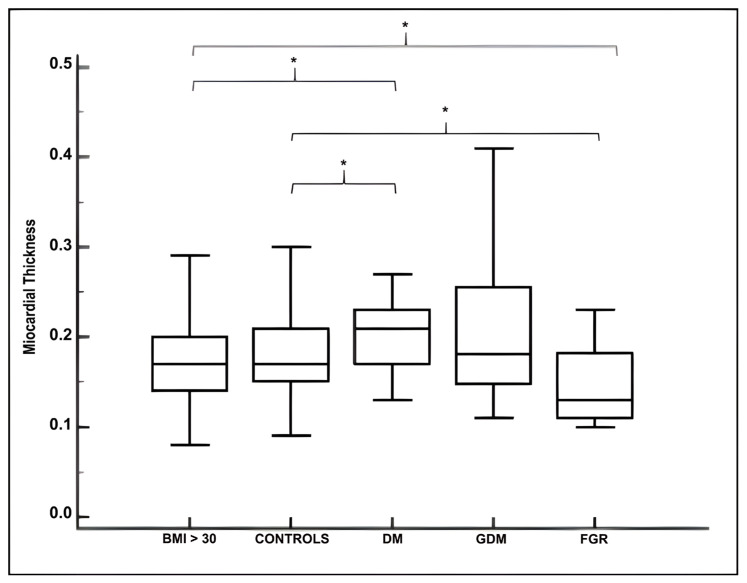
Boxplot charts of myocardial thickness (cm) in normal Controls, BMI group, DM group, GDM group and FGR group, represented as median (IQR). Statistically significant differences between groups are indicated by curly brackets and asterisks (*). BMI: body mass index; DM: diabetes mellitus; GDM: gestational diabetes mellitus; FGR: Fetal Growth Restriction.

**Figure 3:**
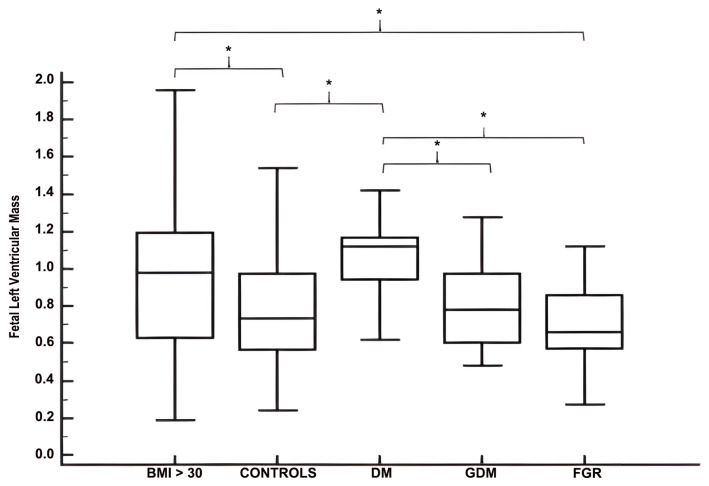
Boxplot charts of fetal left ventricular mass (g) in normal Controls, BMI group, DM group, GDM group and FGR group, represented as median (IQR). Statistically significant differences between groups are indicated by curly brackets and asterisks (*). BMI: body mass index; DM: diabetes mellitus; GDM: gestational diabetes mellitus; FGR: Fetal Growth Restriction.

Table 2 reports the echocardiographic parameters of each group calculated using fetalHQ. Figure 2 and Figure 3 present boxplot charts illustrating myocardial thickness and fetal left ventricular mass for both case and control groups Compared with controls (median: 0.17; IQR: 0.15–0.21), myocardial tissue was thicker in the DM group (median: 0.21; IQR: 0.17–0.23), whereas the FGR group exhibited significantly lower values (median: 0.13; IQR: 0.11–0.18).

When comparing the myocardial thickness among pathological groups (Figure 2), no difference was observed between the DM (median: 0.21; IQR: 0.17–0.23) and GDM groups (median: 0.18; IQR: 0.14–0.25). The myocardial thickness in the BMI group (median: 0.17; IQR: 0.14–0.20) appeared lower than in the DM group but higher than in the FGR group.

Compared with physiological pregnancies (median: 0.73, IQR: 0.56–0.97), fetal left ventricular mass was significantly increased in patients with obesity (BMI group, median: 0.98; IQR: 0.62–1.19) and in the DM group (median: 1.12; IQR: 0.94–1.17). Figure 3 illustrates fetal left ventricular mass across pathological groups and Controls. Values in the DM group were higher than in the GDM (median: 0.78; IQR: 0.60–0.97) and FGR (median: 0.66; IQR: 0.57–0.86) groups. Although the FGR cohort did not differ significantly from controls, their ventricular mass was lower than that of the BMI and DM groups.

Hierarchical testing confirmed the primary findings: KW H = 28.4/35.2 (both *p* < 0.0001); Dunn’s post hoc with Bonferroni’s correction identified DM/FGR (thickness) and DM/BMI (LVM) differences vs. Controls (all *p* < 0.0083) (Table 3).

Finally we used a Multivariable General Linear Model (GLM regression models) adjusted for GA + EFW confirming the robustness of the primary findings (Table 4).

## 4. Discussion

FetalHQ is an emerging tool for advanced echocardiography for speckle-tracking analysis of the fetal heart. The aim of this study was to assess midtrimester morphological alterations in myocardial thickness and left ventricular mass in fetuses from physiological pregnancies and in fetuses exposed to pregravidic maternal diseases such as obesity and DM, and pregnancy-related conditions such as GDM and FGR.

Previous research showed a direct association between maternal obesity or excessive nutrition and cardiac remodeling in offspring. Several studies in both animals and humans have demonstrated a higher ventricular mass and interventricular septal thickness, as well as altered cardiac geometry, in newborns from obese mothers. Specifically, concentric remodeling, increased sphericity index, and mild systolic/diastolic impairment were the most common findings, which, surprisingly, could be mitigated if lifestyle interventions were initiated before or during pregnancy [18,19]. Only limited studies investigated fetal echocardiographic indices in obese mothers, revealing signs of impaired relaxation and possible load-dependent alterations in right ventricular function. In our study, fetuses from the BMI group exhibited thicker myocardial tissue than FGR, while the left ventricular mass was significantly increased compared with both normal Controls and FGR. The mechanisms underlying the association between excessive BMI and the development of hypertrophic cardiomyopathy (HMC) are not fully understood and could be related to multifactorial factors such as placental dysfunction, systemic inflammation and insulin resistance. Experimental models demonstrated that maternal obesity leads to cardiomyocyte hypertrophy, autonomic imbalance, and endothelial dysfunction, supporting a causal pathway from maternal metabolic status to cardiovascular risk in offspring [17].

In pregnancies complicated by DM or GDM, maternal hyperglycemia has been shown to adversely affect fetal cardiac development, but its influence is considered a transient condition with spontaneous regression during the first six months of postnatal life (corresponding to the normalization of insulin levels) [25,36].

An STIC M-mode analysis of fetuses from diabetic mothers highlighted a prevalence of 79.3% of HMC, in which septal hypertrophy was the most prominent alteration, probably because of the high amount of insulin receptors located there [37].

When fetalHQ has been introduced into clinical practice, studies have demonstrated deteriorated fetal cardiac function, as assessed by ventricular fractional shortening (FS), which was significantly reduced in both the left and right ventricles in fetuses of mothers with GDM, especially in the distal ventricular sections [18]. In addition, right ventricular transverse contractility has been shown to be impaired in GDM-exposed fetuses, especially in the mid-apical segments [25]. These results have been recently confirmed by Huang et al., who hypothesized that functional parameters (global longitudinal strain, ejection fraction, and fractional area change) were more sensitive and may change before morphological parameters (global, left and right ventricular spherical index). Moreover, the right ventricle appeared more vulnerable than the left during intrauterine development, whereas the mid-apical sections tended to be affected earlier than the basal ones [23].

Diabetes-associated anatomical and functional modifications are associated with increased perinatal morbidity, as the newborn can suffer from cardiomegaly, respiratory distress secondary to poor left ventricular compliance, and in rare instances, circulatory insufficiency and fetal acidemia. However, the exact mechanisms relating maternal hyperglycemia to abnormalities in fetal cardiac structure and function remain unclear [38,39].

Several authors assumed that the severity of fetal cardiac remodeling could correlate with maternal metabolic control, specifically with HbA1c levels and the duration of diabetes [15,17,19].

These insights suggest that implementing early interventions in the presence of maternal comorbidities may prevent or attenuate fetal myocardial impairment, which can be effectively monitored using advanced echocardiographic techniques. On the contrary, a prospective study by Sapanont et al. demonstrated that, even if HMC was prevalent among diabetic pregnancies, the septal, left and right ventricular thickness were not related to glycemic control [37].

Even a meta-analysis by Depla concluded that diastolic and global cardiac function were effectively impaired on prenatal ultrasound in diabetic pregnancies, irrespective of whether diabetes was pre-gestational or gestational. Moreover, severe HMC occurred even in GDM cases with good glycemic control, advocating that other metabolic factors beyond hyperglycemia contribute to cardiac remodeling [16,37].

In our retrospective analysis, both left ventricular myocardial thickness and mass were significantly increased in cases of maternal diabetes, but only in cases of DM. This may be explained by the fact that exposure to a hyperglycemic environment prior to conception and during the first trimester could be associated with diabetic embryopathy and subsequent consequences for the development of the heart. Early hyperglycemia is well known as a teratogenic agent for cardiac organs, large vessels and neural tubes [40]. In addition, chronic fetal hyperinsulinemia could lead to increased cardiac mass through the enlargement of myocardial nuclear mass, a larger number of cells, and myocardial fiber hypertrophy secondary to enhanced protein and fat synthesis, regardless of the amount of glycogen storage. These changes could be the result of the higher density of insulin receptors in the fetal heart [41]. A link between HMC and high insulin levels in amniotic fluid has also been demonstrated [42]. The histological examination of cardiac tissue from babies of diabetic mothers revealed vacuolization and hydropic modifications of hyperplastic cardiomyocytes, together with increased nuclear and sarcolemmal mass [43].

To the best of our knowledge, our study is the first to focus on myocardial thickness and ventricular mass volume in FGR fetuses. Previous studies have reported fetal cardiac remodeling, characterized by enlarged hearts and a more global sphericity index, as well as abnormalities in cardiac function, including altered myocardial deformation and ventricular contractility [44,45,46]. Aligning with previous research, Xiao et al. scanned a population of FGR fetuses using fetalHQ, demonstrating differences compared with healthy controls in biventricular strain, four-chamber transverse width, as well as in the sphericity index, with the heart having a more globular shape [47]. In our population, the FGR group showed a reduced myocardial thickness in comparison with the obese cohort and Controls, whereas the left ventricular mass had a lower volume than the BMI and DM groups.

We hypothesized that these changes in FGR could be explained by the presence of multiple pathological factors that lead to impaired placental perfusion, chronic hypoxia, increased cardiac workload, and reduced myocardial contractility. In a sheep-based model, Barooni et al. [2] demonstrated that FGR offspring exhibited a lower proportion of cycling cardiomyocytes compared with controls. In addition, these cardiomyocytes preferentially differentiated their cycling toward polyploidization rather than proliferation, revealing a negative linear relationship between cardiomyocyte endoreplication and cardiac mass. These findings suggest that impairments in FGR cardiac growth could be attributed to reduced DNA replication frequency and that cardiomyocyte fate following DNA replication is highly sensitive to adverse intrauterine conditions [47].

Studies in FGR and SGA infants have demonstrated early and persistent alterations in cardiac structure and function, with abnormal myocardial relaxation and reduced overall cardiac performance, likely reflecting increased myocardial workload in utero. Notably, the impact of remodeling was mitigated in infants with longer breastfeeding duration, emphasizing the influence of postnatal nutrition on myocardial development [48,49].

### Future Directions and Potential Applications

This study positions midtrimester fetalHQ as a reliable marker of cardiac remodeling in high-risk pregnancies, warranting prospective longitudinal trials with serial assessments at 18–20, 24–26, and 32+ weeks to delineate progression and correlate with biomarkers (e.g., HbA1c, placental growth factor). Multicenter RCTs should evaluate interventions such as preconception lifestyle optimization in obesity/DM (diet/exercise to avert hypertrophy) and metformin intensification in GDM, using fetalHQ parameters (LV mass, thickness) as primary endpoints alongside neonatal/postnatal follow-up [50,51].

Clinically, fetalHQ enables precision risk stratification: LV mass > 1.1 g or myocardial thickness > 0.20 cm at 20 weeks signals the need for intensified surveillance (biweekly biophysical profiles, uterine artery Doppler per ISUOG guidelines), potentially reducing perinatal cardiomyopathy and long-term CVD risk in FGR offspring. Integration into routine high-risk protocols—alongside AI-enhanced automation—could standardize second-trimester screening, enhancing accessibility beyond expert centers [52].

Future research priorities include: ethnically diverse validation and cost-effectiveness vs. standard biometry assessments; long-term cohort tracking and remodeling to childhood CVD outcomes; and fetal cardiac interventions (e.g., valvuloplasty) guided by fetalHQ in severe cases.

## 5. Conclusions

This study demonstrated that midtrimester echocardiography, improved with advanced speckle-tracking analysis software, can reveal early structural and functional alterations in fetuses exposed to several pregnancy comorbidities. FetalHQ proved to be a valuable instrument for detecting fetal cardiac remodeling, including changes in myocardial thickness and ventricular mass, that are already evident as early as the second trimester. This makes fetal echocardiography a key tool for risk stratification, early follow-up planning, and the optimization of the timing of interventions in high-risk pregnancies, improving long-term neonatal outcomes and postnatal surveillance.

Further research will be needed to explore whether dietary, lifestyle and medical interventions could effectively mitigate fetal cardiac remodeling, and if these benefits can be monitored and optimized through serial echocardiographic follow-ups.

### Limitations and Strengths

This study has several limitations. First, its retrospective design may introduce selection bias, as only pregnancies with technically optimal fetalHQ images (≥80 Hz frame rate, no rib artifacts) were included, potentially underrepresenting cases with suboptimal visualization. Second, all fetalHQ analyses were performed by two experienced operators (A.C., E.C.), which ensures consistency but limits generalizability and introduces potential operator subjectivity despite the software’s semi-automated nature and reported moderate reproducibility. Third, the single-center design restricts ethnic/racial diversity and external validity. Strengths include the largest reported midtrimester fetalHQ cohort (n = 374) across five well-defined groups, consecutive sampling minimizing referral bias, multivariable adjustment for gestational age/EFW, and clinical relevance for high-risk pregnancy risk stratification.

## Figures and Tables

**Table 1:** Maternal biophysical characteristics, gestational weeks at the time of echocardiography and fetal estimated fetal weight. *p*-values were calculated by comparing each case group with the control group. BMI: body mass index; DM: diabetes mellitus; GDM: gestational diabetes mellitus; FGR: Fetal Growth Restriction; EFW: estimated fetal weight.

Characteristics	Controls (n = 108)	BMI Group (n = 119)	*p*	DM Group (n = 69)	*p*	GDM Group (n = 41)	*p*	FGR Group (n = 37)	*p*
Age (years)	34.9 ± 4.4	33.3 ± 6.2	0.027	34.9 ± 4.4	1.0	35.4 ± 5.0	0.551	34.3 ± 4.4	0.475
BMI (kg/m^2^)	24.81 ± 4.17	38.73 ± 4.53	<0.05	22.43 ± 5.22	<0.05	30.98 ± 7.25	<0.05	22.43 ± 5.22	<0.05
Gestational weeks	22.4 ± 3.78	21.5 ± 2.3	0.029	20.4 ± 1.64	0.013	21.4 ± 1.67	0.104	22.7 ± 1.46	0.639
EFW (g)	469.9 ± 127.5	463.9 ± 203.5	0.891	384.6 ± 127.5	0.109	398.5 ± 96.2	0.292	358.3 ± 122.9	0.120

**Table 2:** Fetal echocardiographic characteristics. Measurements via fetalHQ (GE Voluson E10) on 4-chamber view: average end-diastolic LV free wall thickness (24 segments, averaged cardiac cycles); LV mass from endo/epicardial tracings at end-systole/end-diastole. *p*-values were calculated by comparing each case group with the control group. N.S.: Not significant; BMI: body mass index; DM: diabetes mellitus; GDM: gestational diabetes mellitus; FGR: Fetal Growth Restriction.

Characteristics	Controls (n = 108)	BMI Group (n = 119)	*p*	DM Group (n = 69)	*p*	GDM Group (n = 41)	*p*	FGR Group (n = 37)	*p*
Myocardial thickness (cm)	0.17 (0.15–0.21)	0.17 (0.14–0.20)	N.S.	0.21 (0.17–0.23)	<0.01	0.18 (0.14–0.25)	N.S.	0.13 (0.11–0.18)	<0.01
Fetal Left Ventricular Mass (g)	0.73 (0.56–0.97)	0.98 (0.62–1.19)	<0.01	1.12 (0.94–1.17)	<0.001	0.78 (0.60–0.97)	N.S.	0.66 (0.57–0.86)	N.S.

**Table 3:** Dunn’s post hoc with Bonferroni’s correction. (*): significant.

Comparison	Thickness *p* (Dunn)	LVM *p* (Dunn)
DM vs. Ctrl	<0.001 *	<0.001 *
BMI vs. Ctrl	0.12	0.002 *
FGR vs. Ctrl	<0.001 *	0.08
GDM vs. Ctrl	0.45	0.62
*p* < 0.0083 (Bonferroni-corrected)		

**Table 4:** GLM regression models adjusted for GA + EFW.

Outcome	Predictor	β (95% CI)	*p*-Value
Myocardial Thickness	GA (weeks)	−0.002 (−0.008, 0.004)	0.41
Myocardial Thickness	EFW (g)	0.0001 (0.000, 0.0002)	0.09
Myocardial Thickness	DM (vs. Ctrl)	0.038 (0.022, 0.054)	<0.001
Myocardial Thickness	FGR (vs. Ctrl)	−0.042 (−0.062, −0.022)	<0.001
LVM	GA (weeks)	−0.01 (−0.03, 0.01)	0.33
LVM	EFW (g)	0.001 (0.0005, 0.0015)	<0.01
LVM	DM (vs. Ctrl)	0.37 (0.18, 0.56)	<0.001
LVM	BMI (vs. Ctrl)	0.25 (0.10, 0.40)	<0.01

## Data Availability

The data presented in this study are available on request from the corresponding author.
